# PDZRN4 suppresses tumorigenesis and androgen therapy-resistance in prostate cancer

**DOI:** 10.7150/jca.69269

**Published:** 2022-04-18

**Authors:** Peng Jin, Lielin Wu, Gang Zhang, Bo Yang, Bisong Zhu

**Affiliations:** Organ Transplant Center, Xiangya Hospital, Central South University, Changsha, Hunan, PCR, 410008.

**Keywords:** PDZRN4, prostate cancer, CRPC, androgen

## Abstract

**Background:** PDZRN4 (PDZ domain-containing RING finger 4), a member of the LNX (ligand of numb protein-X) family that regulates the levels of NUMB, plays a critical role in suppressing the proliferation and invasion of hormone-related malignant tumours. There are few studies on the role of PDZRN4 in the pathogenesis of prostate cancer (PCa). We aimed to examine whether PDZRN4 regulates the growth and development of PCa.

**Methods:** Cell transduction and Western blotting were used to establish and confirm PDZRN4 knock down in PC cells. Using the MTT, wound healing, transwell migration, and animal experiments, we explored the biological function of PDZRN4 knockdown (PDZRN4-kd) cells. Via PCR and immunohistochemistry, the mRNA and protein expression of PDZRN4 was examined in PC cells and tissues.

**Results:** Hormone-dependent (LNCap) and hormone-independent (DU145, PC3, and C4-2) PC lines were transfected with lentivirus carrying PDZRN4 shRNA. The Western blotting results showed that the expression of PDZRN4 was stably downregulated in PDZRN4 knockdown (PDZRN4-kd) cells. The proliferation, invasion and migration of PDZRN4-kd cells were dramatically increased *in vivo*. To explore the expression of PDZRN4 in prostate cancer samples, we analysed TCGA data and found that PDZRN4 was negatively correlated with the development of PC. PDZRN4 levels were downregulated by androgen deprivation in hormone-sensitive cells. Moreover, PDZRN4 failed to induce proliferation in DU145 cells with androgen deprivation.

**Conclusions:** PDZRN4 is a functional suppressor of prostate cancer growth and development and is a potential target of biochemical therapy in hormone-resistant PC.

## Introduction

Prostate cancer (PCa) is the most common cancer in men in the United States and Europe 1, 2, and the incidence and mortality rates of PC have also dramatically increased over the past two decades in developing countries such as China and Brazil 3. In China, the incidence of PC has increased at an annual rate of 12.1%, resulting in approximately 60,300 new cases in 2015 4. Androgen plays a critical role in prostate gland development and physiologic function. The androgen receptor (AR) in untreated PC tissue remains highly expressed and is a target of chemotherapy 5. However, all metastatic PC cases gradually develop resistance to hormone-targeted therapy and transform into castration-resistant prostate cancer (CRPC) within 1-2 years 6. To explore the mechanism involved in resistance to hormone inhibitors in CRPC treatment, an increasing number of studies have focused on the differentiating characteristics between androgen-sensitive PC and CRPC 7.

PDZRN4 (PDZ domain-containing RING finger 4) is a member of the LNX (ligand of numb protein-X) family and is located on human chromosome 12. Members of this family regulate the levels of NUMB by acting as E3 ubiquitin ligases that specifically target NUMB for ubiquitin-dependent proteasome degradation 8, 9. Previous studies have demonstrated that PDZRN4 plays a critical role in suppressing proliferation and invasion in malignant tumours 10, 11. Nevertheless, there are few studies on the role of PDZRN4 in prostate cancer. In this study, we demonstrate that PDZRN4, acting as a cancer-suppressing gene, reduces the proliferation, invasion and migration capacities of PC cells regulated by androgen activation.

## Methods

### Cell lines and reagents

The prostate cancer cell lines DU145, C4-2, PC3, and LNCap were purchased from ATCC. DU145 and LNCap cells were cultured with RPMI 1640 (BI, USA) mixed with 10% FBS. The anti-PDZRN4 antibody (ab171083) was purchased from Abcam, USA. The shRNA targeting PDZRN4 (ID: NM_013377.2-3072s1c1), lentivirus vector, and transduction reagent, PEI (polyetherimide) was purchased from Sigma, USA.

### Transduction

PC cells were transduced with a mix of PEI and shRNA-carrying lentivirus. After 48 hours, the cells were collected and lysed for subsequent Western blotting. A total of 1×10^4^ transfected cells were reseeded into 10 cm dish cultured with media including puromycin. Fifteen days later, the cell colonies were respectively picked into 96-wells and collected for cell lysis.

### Western blotting

The cell lysis buffer for cell protein collection included 50 mM Tris HCl, 250 mM NaCl, 3 mM EDTA, 3 mM EGTA, 1% Triton, 0.5% NP40, 10% glycerol, 2 mM DTT, 1 mM PMSF, 0.1 mM Sodium vanadate, 2 mM PNPP, and 1:1000 Proteases inhibitors. Thirty to fifty μg protein per well was loaded on12% acrylamide gels. PVDF membranes were washed by TBST with 0.1% Tween, and were then incubated with the anti-PDZRN4 antibody (1:1000, diluted with 5% BSA) for 16 hours at 4 °C. The membranes were washed with PBS three times and incubated with secondary rabbit antibody (1:100,000) for 2 hours at room temperature. They were then developed by ECL, using ChemiDoc^TM^ XRS+ Imaging System (BIO-RAD Molecular Imager).

### MTT assays

Ten thousand cells per well were grown in 96-well plates and cultured in normal medium/charcoal medium for 1-4 days. At the checkpoint, 10 μl of MTT was added per well, and the plates were incubated at 37 °C for 3 hours. The original medium was then removed, and 100 μl DMSO was added. A microplate reader (Tecan, USA) was used to measure the spectrometric absorbance at 570 and 630 nm.

### Wound healing

Cells were inoculated in 6-well plates and cultured overnight. When 90% confluence was reached, the cells were scratched with 10-μl tips. Cell images were captured using an inverted microscope at 0, 24 and 48 hours.

### Transwell migration assays

The upper well of transwell system was loaded by 50 μl FBS-free medium with 20% Matrigel (Corning, USA). The upper chambers were filled with 5×10^4^ cells in RPMI 1640 medium with 1% FBS, and the lower chambers were filled with medium with 10% FBS as a chemoattractant. The cells invading through the Matrigel were stained by crystal violet and visualized by inverted microscopy.

### Animal experiments

Four-week-old male nude mice (Balb/c background) were chosen for animal experiments. Before injecting the cells using injection syringe, we removed the hair on the mice back by depilatory cream and disinfect the skin with 75% alcohol. Approximately 2×10^6^ suspended PDZRN4-KD or control LNCap or PC3 cells mixed at a 1:5 ratio with Matrigel medium were injected percutaneously into subcutaneous tissue of the backs of mice. Four mice were in each group. After 4 weeks, the mice were killed, and the tumours were collected. The mice were sacrificed by high concentrations of CO_2_. The weight and volume of the tumours were measured. This animal xenograft study was approved by the Research Ethics Committee of Xiangya Hospital.

### Quantitative real-time PCR

cDNA was synthesized using 1 μg of the total RNA extracted by TRIzol (Invitrogen, USA) and the TaqMan reverse transcription reagent. The primers for PDZRN4 were as follows: 5′- CCAGCACTCAGACGGACATC-3′ and 5′-CTAACACGACACAACTCGACC-3′; the primers for GAPDH were as follows: 5′-GGAGCGAGATCCCTCCAAAAT-3′ and 5′- GGCTGTTGTCATACTTCTCATGG-3′. The relative levels of target gene mRNAs were expressed relative to those of β-actin and calculated from the standard curve according to the manufacturer's instructions. The 2xSG Fast qPCR Master Mix (High Rox, BBI LIFE SCIENCES CORPORATION, China) was used for qPCR, by CFX Connect^TM^ Real-Time System (BIO-RAD, USA).

### PC patient enrolment and immunohistochemistry

From July 2017 to December 2019, 52 PC patients not yet receiving treatment and 37 CRPC patients admitted to Xiangya Hospital, Central South University, were enrolled. This study was approved by the Research Ethics Committee of Xiangya Hospital.

The PC biopsy tissues were preserved in formalin. The samples were cut into 5-um thick paraffin sections. Immunohistochemical (IHC) staining for PDZRN4 (1:200) was performed on tissue slides. Six areas under 200× magnification were randomly chosen for IHC scoring of each slide. The staining results were simultaneously evaluated by two independent pathologists (double-blinded).

### Statistical analysis

All experiments were performed at least three times, and the corresponding variables are expressed as x ± σ. Statistical analyses were performed using single and two-tailed t-tests and Chi-square test. The statistical significance of the observed differences was determined according to the p-value (* p < 0.05, ** p < 0.01). Kaplan-Meier survival curves were plotted, and the number-at-risk was indicated below the main plot. Hazard ratio (HR), 95% confidence intervals (CI), and log-rank *p* values were calculated and displayed.

## Results

### PDZRN4 suppresses proliferation, migration and invasion in prostate cancer

**Figure 1:**
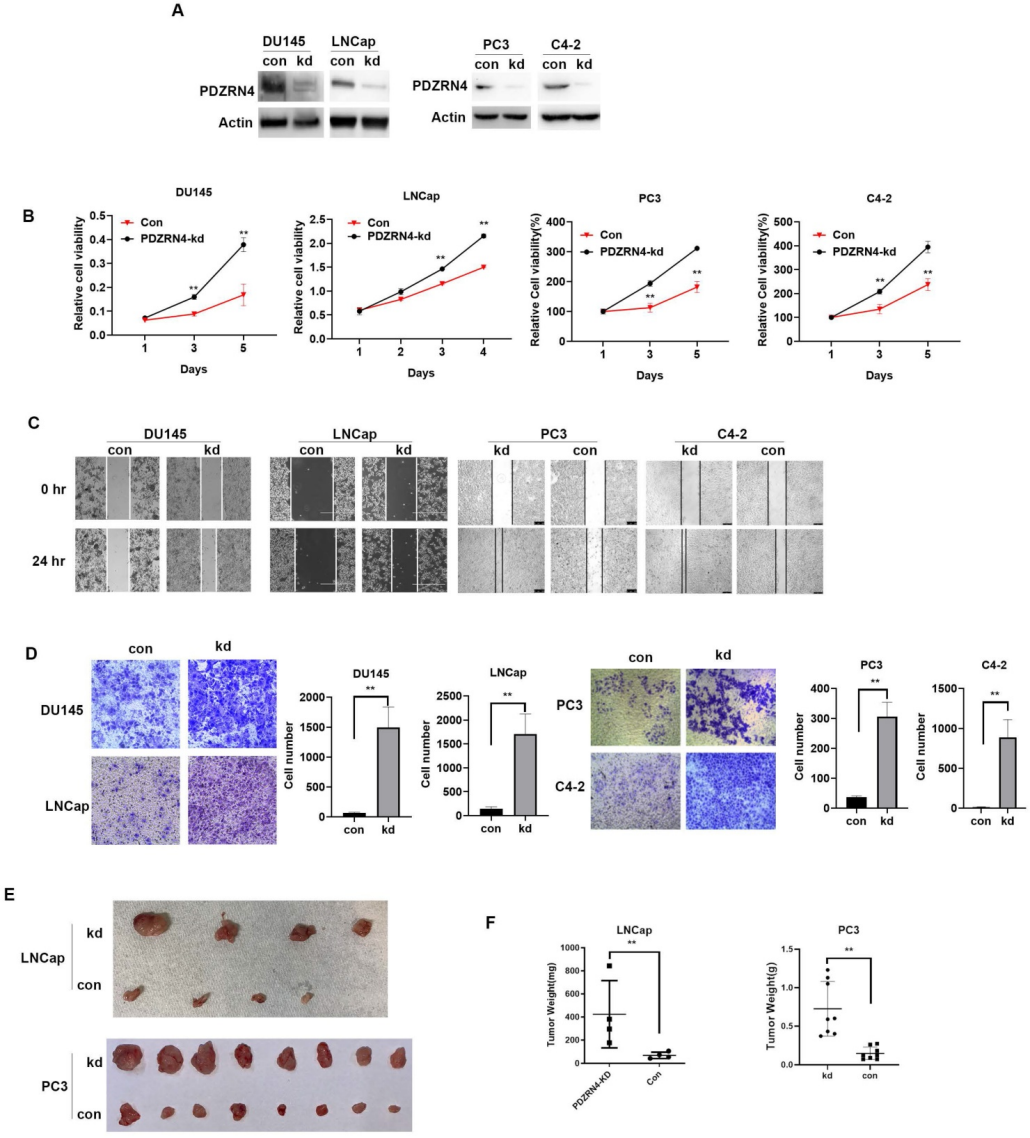
** PDZRN4 suppressed the proliferation, migration and invasion in prostate cancer. A.** Western blotting showing PDZRN4 expression in DU145, PC3, C4-2 and LNCap cells transfected by PDZRN4 shRNA lentivirus (kd) or control (con). **B.** MTT assay showing the proliferation of DU145, PC3, C4-2 and LNCap cells transfected by PDZRN4 shRNA lentivirus (PDZRN4-kd) or control (Con). **C.** Wound healing analysis of DU145, PC3, C4-2 and LNCap cells transfected by PDZRN4 shRNA lentivirus (kd) or control (con). **D.** Transwell assays of DU145, PC3, C4-2 and LNCap cells transfected by PDZRN4 shRNA lentivirus (kd) or control (con). **E, F.** Xenograft tumor experiment in nude mice injected with LNCap and PC3 cells stably transfected by PDZRN4 shRNA lentivirus (kd) or control (con).

Lentivirus carrying the shRNA or empty vector was transfected into PC DU145, PC3, C4-2 and LNCap cells. Western blotting showed that the levels of PDZRN4 were decreased in PDZRN4-knockdown (PDZRN4-kd) DU145, PC3, C4-2 and LNCap cells (Figure 1A). Using MTT, wound healing and transwell assays, we found that PDZRN4-kd cells had significantly induced cell proliferation (Figure 1B and SF B), migration (Figure 1C) and invasion (Figure 1D) compared with the respective control cells. We injected stably transfected PDZRN4-kd LNCap and PC3 cells into 4-week-old nude mice for xenograft experiments. After 4 weeks of growth, the tumour weights in mice injected with PDZRN4-kd LNCap and PC3 cells were significantly higher than those of in mice injected with control cells (Figure 1E, F). These results show that PDZRN4 reduced the growth and development of PC *in vitro* and *in vivo*.

### The expression of PDZRN4 in the TCGA database

**Figure 2:**
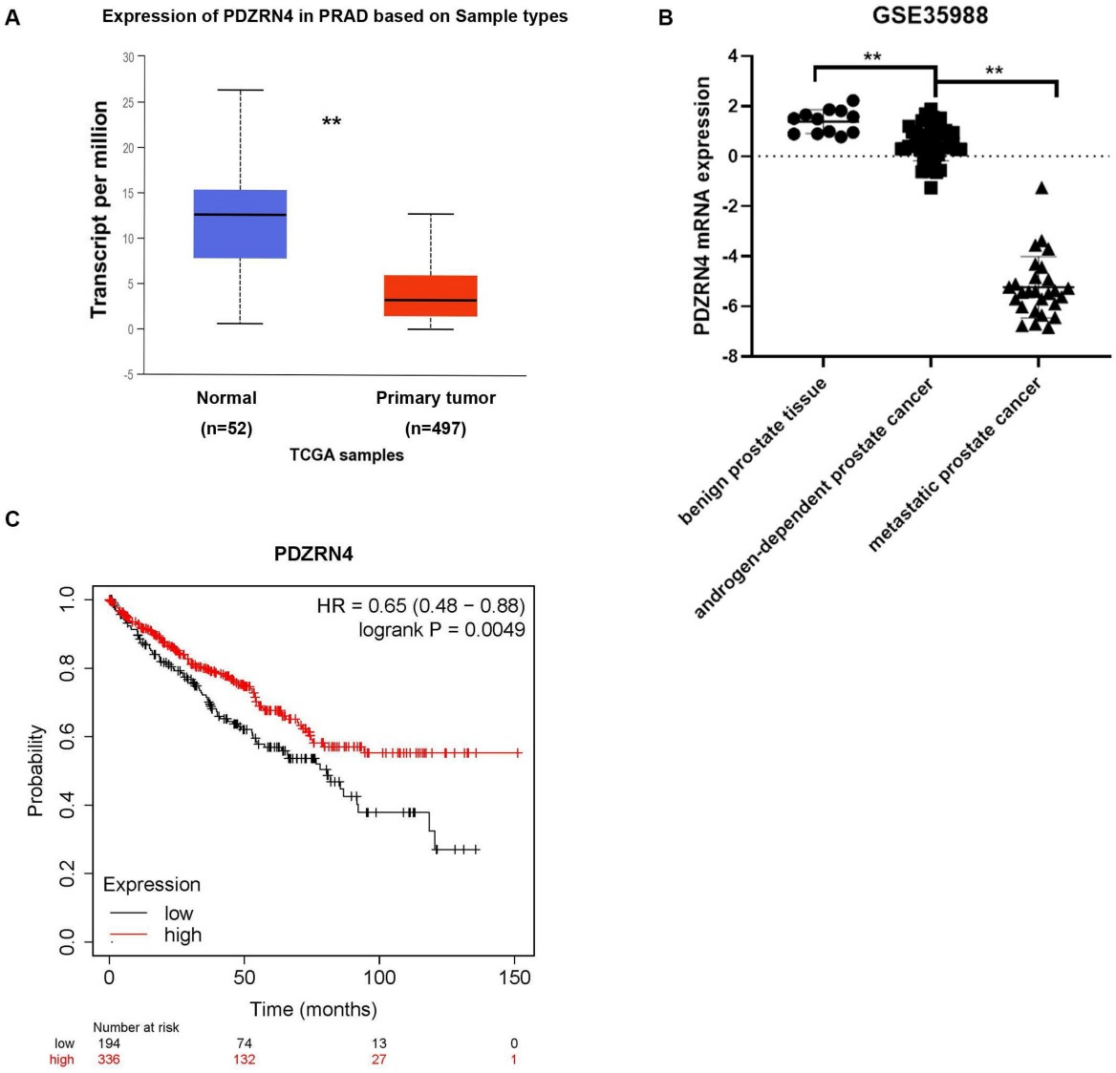
** The expression of PDZRN4 in TCGA database. A.** The expression of PDZRN4 in prostate cancer and normal prostate tissues (http://ualcan.path.uab.edu/analysis.html). **B.** The expression of PDZRN4 in metastatic prostate cancers, localized prostate cancers and benign prostate tissues (https://www.ncbi.nlm.nih.gov/sites/GDSbrowser). **C.** Kaplan-Meier survival curves comparing overall survival rates on the basis of high and low expression of PDZRN4 in prostate cancer patient cohort (https://www.kmplot.com/).

To detect the expression of PDZRN4 in prostate cancer tissues, we explored several datasets in the TCGA system. The mRNA levels of PDZRN4 in PC were sharply downregulated compared with those in normal prostate tissues (Figure 2A). To analyse the difference in PDZRN4 expression between benign prostate, androgen-dependent PC and CRPC tissues, the GSE35988 dataset was downloaded from the GPL9075 platform (https://www.ncbi.nlm.nih.gov/sites/GDSbrowser). It was demonstrated that the relative mRNA levels of PDZRN4 in CRPC were dramatically lower than those in androgen-dependent PCa, while PDZRN4 was overexpressed in benign tissue (Figure 2B). Kaplan-Meier survival analysis showed that the PC patients with low expression of PDZRN4 had a low survival rate (Figure 2C, *P*=0.0049). These data illustrate that PDZRN4 had a negative correlation with proliferation and invasion in PC and that androgen may induce the expression of PDZRN4.

### Androgen deprivation reduced the expression of PDZRN4

**Figure 3:**
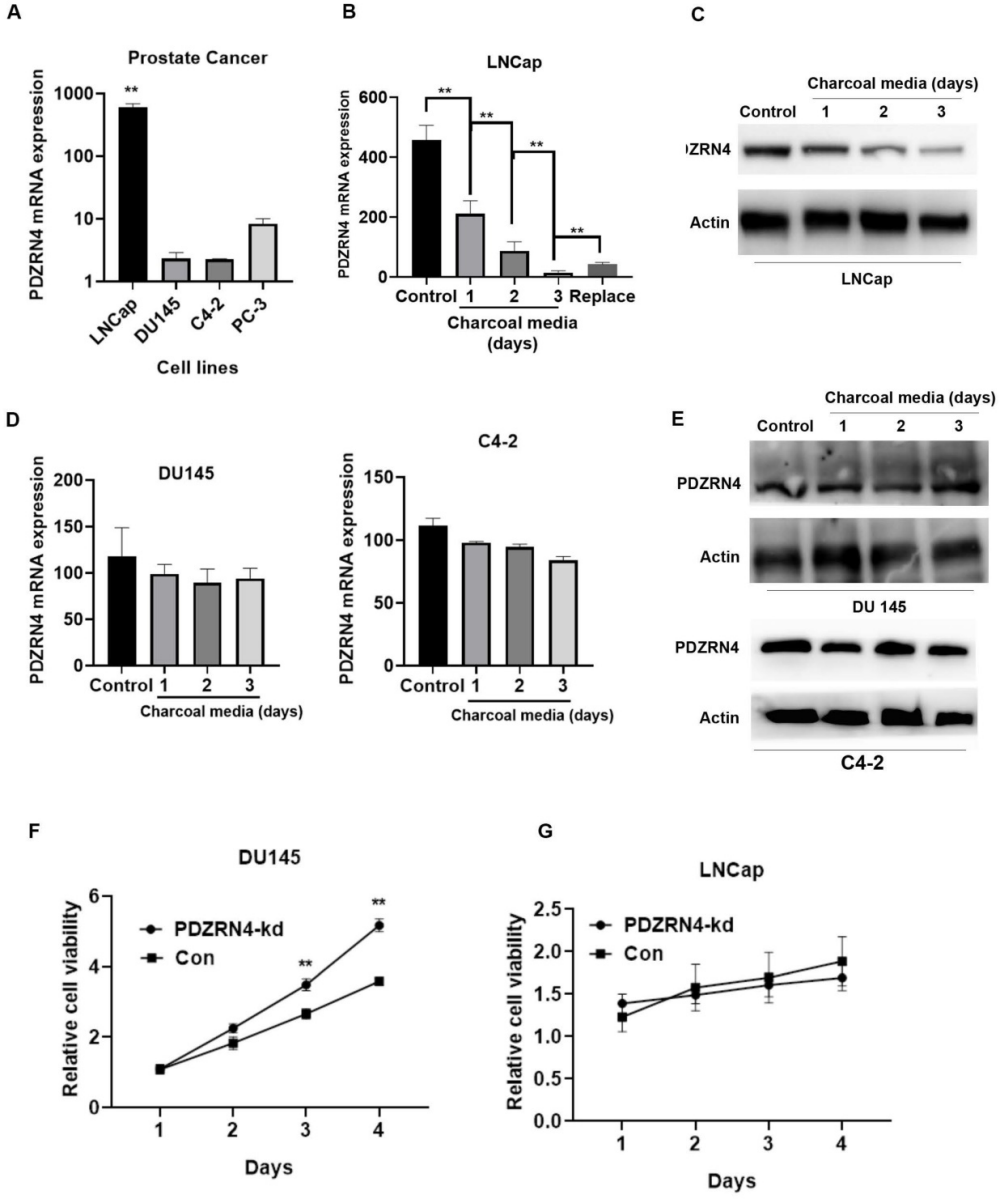
** PDZRN4 was decreased and lost the ability of reducing proliferation in androgen-dependent cells (LNCap) upon androgen deprivation. A.** qRT-PCR assays of the PDZRN4 mRNA expression of PC cell lines LNCap, DU145, C4-2, and PC-3. **B.** qRT-PCR assays of the PDZRN4 mRNA expression of LNCap cell treated by charcoal media for gradient days or by normal media (Control). **C.** Western blotting showing the PDZRN4 levels of LNCap cell treated by charcoal media for gradient days or by normal media (Control). **D.** qRT-PCR assays of the PDZRN4 mRNA expression of DU145 and C4-2 cell treated by charcoal media for gradient days or by normal media (Control). **E.** Western blotting showing PDZRN4 levels of DU145 and C4-2 cell treated by charcoal media for gradient days or by normal media (Control). **F, G.** MTT assays displaying the proliferation of LNCap and DU145 cell transfected by PDZRN4 shRNA lentivirus (PDZRN4-kd) or control (Con) cultured by charcoal media.

To examine the effect of androgen on PDZRN4, the mRNA expression of PDZRN4 in variant PC cell lines were first evaluated by qRT-PCR. The results showed that the level of PDZRN4 in LNCap, an androgen-dependent PC cell line, was dramatically higher than that in androgen-resistant PC cell line. Then, we cultured androgen-resistant PC cells (DU145) and androgen-dependent PC cells (LNCap), in charcoal media (media without hormones). We found that the mRNA and protein expression of PDZRN4 was significantly decreased in LNCap cells cultured in charcoal media (Figure 3B and C) and re-increased as replaced normal media, while there was no significant difference in DU145 and C4-2 cells (Figure 3D and E). The MTT assay showed that the rate of proliferation in PDZRN4-kd DU145 cells was higher than that in control cells cultured in charcoal media (Figure 3F). However, when LNCap cells were cultured in charcoal media, PDZRN4 knockdown failed to activate tumour growth (Figure 3G). These results demonstrated that PDZRN4 failed to regulate the growth of hormone-sensitive PC cells without androgen participation, but the expression and function of PDZRN4 were independent of androgen influence in hormone-resistant PC cells.

### PDZRN4 levels were correlated with clinicopathologic characteristics in PC patients

**Figure 4:**
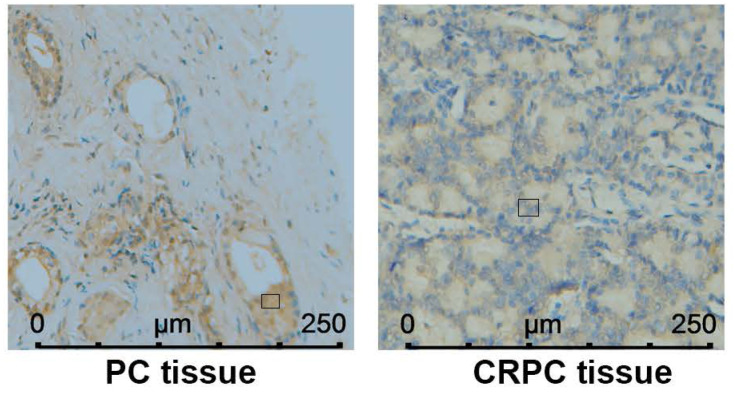
PDZRN4 expressed in androgen-dependent PC and CRPC by immunohistochemistry.

In Table 1, the value of PSA (prostate-specific antigen) was positively corelated with PDZRN4 staining. The protein expression of PDZRN4 in CRPC tissues was remarkably decreased compared with the level in PC tissues (Figure 4, Table 2). There was a strong correlation between PDZRN4 and clinicopathologic characteristics in PC patients.

## Discussion

PDZRN proteins comprise a protein family with similar RING finger domains and variable PDZ domains. It has been confirmed that PDZRN4 downregulates E3 ubiquitin ligase activity 12, 13. PDZRN3 and PDZRN4 share identical structures consisting of one RING finger domain and two PDZ domains 14. PDZRN3 plays an essential role in the differentiation of head and neck tumour cells 15, 16. Hu et al. showed that ectopic expression of PDZRN4 inhibits proliferation, plate colony formation and anchorage-independent colony formation in HCC cells 10.

In this study, we illustrated that PDZRN4 reduces the growth and invasion of PC cells *in vitro* and* in vivo*. Our *in silico* analysis demonstrated that the expression of PDZRN4 was suppressed during PC development. PDZRN4 levels were downregulated by androgen deprivation in hormone-sensitive cells. In PC patients, the expression of PDZRN4 was correlated with clinicopathologic characteristics, such as TNM stage and differentiation.

We also found that the levels of PDZRN4 were decreased in prostate cancer, especially in CRPC tissues (Figure 2B). Therefore, four PC cell lines, androgen-resistant DU145, PC3, and C4-2 cells and androgen-dependent LNCap cells, were chosen for further experiments examining the function of PDZRN4. Our study confirms that PDZRN4 suppresses the growth and invasion of PC cells *in vivo* and *in vitro*. In particular, the size of PDZRN4-kd tumours was dramatically larger than that of control tumours.

Interestingly, when cultured in charcoal media, a method to temporarily induce hormone deprivation, the expression of PDZRN4 in LNCap cells was dramatically decreased compared with that in cells cultured with regular media, while PDZRN4 expression in DU145 cells remained stable regardless of hormone availability. In androgen-dependent cells, proliferation is regulated by androgen activation (Figure 3). Yu et al. found that tumour proliferation was suppressed by androgen in LNCap-AI cells, which led to cell cycle arrest at the G1 phase. Conversely, androgen significantly increased LNCaP cell proliferation by promoting the G1-S transition 17. In breast cancer, RAD140 is a potent AR agonist in cells with a distinct mechanism of action, and AR-mediated repression of ESR1 has been documented 18, 19. The growth of tumours in multiple AR/ER+ breast cancer PDX models can be induced by AR agonists combined with palbociclib 20. Endometrial androgen biosynthesis and intracrine action are important in the development of a tissue microenvironment that can support implantation and establishment of pregnancy 21. Androgens on endometrial cell proliferation participate in repairing the endometrial wound at the time of menstruation and during endometrial disorders 22.

In clinical practice, the first-line therapy-strategy of CRPC typically targets AR signalling pathway. The therapeutic effects tend to rapidly resolve resulting from the CRPC addiction to AR-mediated transcription 23. Despite the advancing anti-androgen treatment, 20%-40% of CRPC patients with metastasis demonstrate *de novo* resistance to the FDA-approved drugs abiraterone and enzalutamide 24. Therefore, the identification and therapeutic targeting should be considered as alternative or complementary strategies to block AR transcriptional addiction and postpone the occurrence of androgen-resistance. In this study, we have demonstrated the interesting phenomenon that PDZRN4, as an androgen-dependent or -independent suppressor, reduces the growth and invasion of PC cells. In androgen-dependent PC cell LNCap, the PDZRN4 level was dramatically decreased with hormone deprivation. While in DU145 cell, the expression and function of PDZRN4 was independent from hormone absence. The uncertain mechanism might be regulated by abnormally activated androgen receptor from non-hormone signalling in androgen-independent PC cells. We plan to explore the co-activation between PDZRN4 and androgen and the mechanism that PDZRN4 independently suppresses CRPC in the future.

## Conclusion

The present study found that the expression of PDZRN4 in PC was negatively correlated with the survival rate. In particular, the levels of PDZRN4 were decreased in CRPC samples. PDZRN4 is a functional suppressor of prostate cancer growth and development and a potential target of biochemical therapy in hormone-resistant PC.

## Figures and Tables

**Table 1:** Correlation between the staining of PDZRN4 and clinicopathologic characteristics in PC tissues

	n	PDZRN4		
		-	+	*P*
**Age(years)**				
≤60	31	14	17	0.214
>60	58	19	39	
**PSA (ng/ml)**				
<10	24	18	6	<0.01
≥10	65	16	49	
**Histologic type**				
Poorly and undifferentiated	37	24	13	0.08
Well and moderate	52	24	28	

**Table 2:** Correlation of PDZRN4 staining between PC tissues before treatment and CRPC tissues (Chi-square test)

	n	PDZRN4			
		-	+	Chi-square, df	*P*
PC before treatment	52	14	38	14.51, 1	0.0001
CRPC	37	25	12		

## Abbreviations

PCa: prostate cancer
PDZRN4: PDZ domain-containing RING finger 4
LNX: ligand of numb protein-X
AR: androgen receptor
CRPC: castration-resistant prostate cancer
PEI: polyetherimide
IHC: immunohistochemistry
PSA: prostate-specific antigen
